# Indoxyl Sulfate Induces Apoptosis through Oxidative Stress and Mitogen-Activated Protein Kinase Signaling Pathway Inhibition in Human Astrocytes

**DOI:** 10.3390/jcm8020191

**Published:** 2019-02-05

**Authors:** Yi-Ting Lin, Ping-Hsun Wu, Yi-Chun Tsai, Ya-Ling Hsu, Han Ying Wang, Mei-Chuan Kuo, Po-Lin Kuo, Shang-Jyh Hwang

**Affiliations:** 1 Institute of Clinical Medicine, College of Medicine, Kaohsiung Medical University, Kaohsiung 807, Taiwan; 960254@kmuh.org.tw (Y.-T.L.); 970392@kmuh.org.tw (P.-H.W.); lidam65@yahoo.com.tw (Y.-C.T.); kuopolin@kmu.edu.tw (P.-L.K.); 2 Faculty of Medicine, College of Medicine, Kaohsiung Medical University, Kaohsiung 807, Taiwan; mechku@kmu.edu.tw; 3 Department of Family Medicine, Kaohsiung Medical University Hospital, Kaohsiung 807, Taiwan; 4 Division of Nephrology, Department of Internal Medicine, Kaohsiung Medical University Hospital, Kaohsiung 807, Taiwan; a334616@yahoo.com.tw; 5 Faculty of Renal Care, College of Medicine, Kaohsiung Medical University, Kaohsiung 807, Taiwan; 6 Graduate Institute of Medicine, College of Medicine, Kaohsiung Medical University, Kaohsiung 807, Taiwan; yainghsu@kmu.edu.tw

**Keywords:** indoxyl sulfate, uremic toxins, astrocyte, oxidative stress, mitogen-activated protein kinase, dual specific phosphatase

## Abstract

Uremic toxins accumulated in chronic kidney disease (CKD) increases the risk of cognitive impairment. Indoxyl sulfate (IS) is a well-known protein-bound uremic toxin that is correlated with several systemic diseases, but no studies on human brain cells are available. We investigated the effect of IS on primary human astrocytes through next-generation sequencing and cell experiment confirmation to explore the mechanism of IS-associated brain damage. Total RNAs extracted from IS-treated and control astrocytes were evaluated by performing functional and pathway enrichment analysis. The toxicities of IS in the astrocytes were investigated in terms of cell viability through flow cytometry; the signal pathway was then investigated through immunoblotting. IS stimulated the release of reactive oxygen species, increased nuclear factor (erythroid-derived 2)-like 2 levels, and reduced mitochondrial membrane potential. IS triggered astrocyte apoptosis by inhibiting the mitogen-activated protein kinase (MAPK) pathway, including extracellular-signal-regulated kinase (ERK), MAPK/ERK kinase, c-Jun N-terminal kinase, and p38. The decreased ERK phosphorylation was mediated by the upregulated dual-specificity phosphatase 1, 5, 8, and 16. In conclusion, IS can induce neurotoxicity in patients with CKD and the pathogenesis involves cell apoptosis through oxidative stress induction and MAPK pathway inhibition in human astrocytes.

## 1. Introduction

Patients with chronic kidney disease (CKD) exhibit higher rates of cognitive impairment and dementia than does the general population [1]. Dementia comorbidity worsens the adverse outcomes, such as disability, hospitalization, and mortality [2,3]. CKD patients are susceptible to more nephrogenic risk factors for dementia than is the general population [4,5]. The major causes of cognitive impairment in patients with CKD include cerebrovascular disease, anemia, secondary hyperparathyroidism, dialysis disequilibrium, and uremic toxins [4,5]. Uremic toxins are accumulated in CKD patients, and they cause various types of damage to the brain, thus interfering with cognitive function [6,7].

CKD is associated with uremic toxin accumulation within the brain tissue [8], and high toxin concentrations in the brain regions play an important role in cognition [9]. Organic anion transporters (OAT3) are responsible for transporting protein-bound uremic toxins, such as indoxyl sulfate (IS) [10]. OATs are expressed in a broad range of organs in living organisms, including the blood–brain barrier (BBB) [11]. IS accumulation within brain structures may be linked to the expression of OAT3 efflux transporter [12,13] in the BBB and the blood–cerebrospinal fluid barrier (BCSFB). Both in vivo and in vitro studies have suggested that infiltration of uremic toxins within brain structures may have deleterious impacts on brain resident cells, such as microglia, astrocytes, and neurons.

Dialysis removes small water-soluble uremic toxins, but has no effect on middle molecules or protein-bound uremic toxins. Because of their strong protein-binding abilities, hemodialytic removal of protein-bound compounds, such as IS, is difficult. High serum IS levels are associated with poorer cognitive function in the early CKD stages [14]. IS induces reactive oxygen species (ROS) production and reduces cell viability in cultured mouse brain endothelial cells [15]. Moreover, IS promotes inducible nitric oxide synthase (iNOS) and cyclooxygenase-2 (COX-2) expression, along with TNF-alpha and IL-6 release and nitrotyrosine formation in primary mouse astrocytes and mixed glial cells [16]. IS has also been reported as an endogenous ligand for AhR [17,18,19], expressed in the brain, including the cerebral cortex, hippocampus, and cerebellum [20]. Cytosolic AhR can activate nuclear factor (erythroid-derived 2)-like 2 (NRF2), a transcription factor that protects against oxidative stress [21]. However, no study has investigated the effect of IS on human astrocytes. Astrocytes are the most abundant glial cells in the central nervous system (CNS), participating in diverse functions, including maintaining the BBB, as well as regulating metabolism, neuronal transmission, CNS development, and inflammation [22]. Moreover, astrocytes alter neuronal functions and modulate neuronal activity indirectly [23]. Therefore, astrocyte dysfunction may contribute to several disease mechanisms [22]. Astrocytes are related to various forms of dementia [24,25], so the potential link between IS and neurotoxicity via astrocyte damage may partially explain the pathophysiology of uremic toxins on cognitive functions in CKD.

In the present study, the authors explored the differentially expressed transcriptomes in IS-treated human astrocytes and identify potential pathophysiology pathway inference and the related network involved in IS-related toxicity.

## 2. Materials and Methods

**Figure 1:**
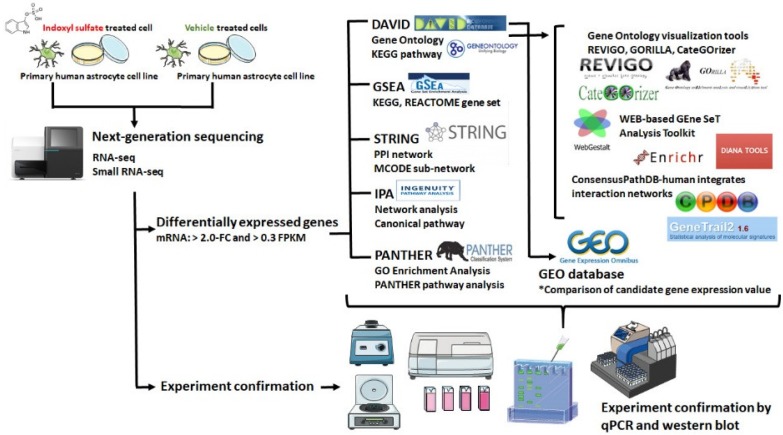
Flowchart of study design. The primary human astrocytes with and without IS treatment were cultured and harvested for RNA sequencing and expression profiling. Differentially expressed genes with >2-fold change (FC) and >0.3 fragments per kilo base of transcript million (FPKM) were selected for further enrichment analyses by using different bioinformatics resources. Array data related to IS-treated cell lines were searched in the Gene Expression Omnibus (GEO) database, and the expression patterns of candidate genes of interest in these arrays were analyzed. The bioinformatics analysis results were further verified by experimental confirmation.

A flowchart of our study design is illustrated in Figure 1.

### 2.1. Cell Culture and Cell Viability

Human primary astrocytes obtained from Lonza (Walkersville, MD, United States of America [USA]) were cultured in astrocyte growth medium ((CellApplications, Cat. No. 821-500, San Diego, CA, USA), according to the manufacturer’s recommendations. Cells from the third passage were cultured in a 10-cm dish and subsequently transferred to 96-well culture plates (2.5 × 10^3^/well) for treatment with uremic solute IS (Sigma-Aldrich, CAS No. 2642-37-7) at different concentrations for 48 h. Cell viability was determined by Water-Soluble Tetrazolium Salt-1 (WST-1) Cell Proliferation Assay (Clontech Laboratories, Mountain View, CA, USA) to measure the mitochondrial desydrogenase activity of living cells.

### 2.2. RNA Sequencing

Total RNA was extracted using Trizol Reagent (Invitrogen, Carlsbad, CA, USA) from normal and IS-treated human astrocytes, and the extract passed the RNA quality control for sequencing. The quality and integrity of total RNA was assessed by performing OD260/OD280 absorbance ratio detection by using a ND-1000 spectrophotometer (Nanodrop Technology, Wilmington, CA, USA). The samples were submitted to Welgene Biotechnology Company (Welgene, Taipei, Taiwan) for sequencing analysis. All procedures for RNA-seq were carried out according to the manufacturer’s protocol from Illumina. The library was constructed using the Illumina Solexa sequencing platform with a read length of 75 nucleotides single-end sequencing. Raw sequences were analyzed, and the low-quality data were filtered out to obtain qualified reads by using the TopHat/Cufflinks method [26]. The gene expression level was calculated in terms of fragments per kilobase of transcript per million mapped reads (FPKM), and the FPKM threshold was set to more than 5 for RNA-seq by considering the balance between the numbers of false positive and false negative detections and higher confidence in the measured expression level [27,28]. The criterion for differentially expressed mRNAs was set to a fold change of more than 2.0.

### 2.3. Bioinformatics Analysis

#### 2.3.1. Functional and Signaling Pathway Analyses

Functional and signaling pathway analyses of the IS-treated astrocytes were performed using a public database platform called the Database for Annotation, Visualization and Integrated Discovery (DAVID; https://david.ncifcrf.gov/) [29]. DAVID provides a functional interpretation of massive gene lists derived from genomic studies. To obtain more information, the Enrichr method (http://amp.pharm.mssm.edu/Enrichr/) [30], Gene Ontology (GO) enRIchment anaLysis and visuaLizAtion (Gorilla; http://cbl-gorilla.cs.technion.ac.il/) [31], and WEB-based Gene SeT AnaLysis Toolkit (WebGestalt; http://www.webgestalt.org/option.php) [32] were used to confirm GO (http://www.geneontology.org/) functionally [33]. Kyoto Encyclopedia of Genes and Genomes (KEGG; http://www.genome.jp/kegg/) [34], Protein ANalysis THrough Evolutionary Relationships (PANTHER; http://www.pantherdb.org/), and BioCarta (http://www.biocarta.com/) were investigated for pathway enrichment analyses. Moreover, pathway analysis and functional annotation were performed using ConsensusPathDB (CPDB; http://cpdb.mplgen.mpg.de) [35] and Ingenuity Pathway Analysis (IPA, QIAGEN Redwood City; www.qiagen.com/ingenuity).

#### 2.3.2. Data Visualization

The cellular component and biological process annotations were classified into broad groups based on the GO-slim classification system by using CateGOrizer (https://www.animalgenome.org/tools/catego/) for visualization analysis [36]. Furthermore, the CateGOrizer outputs were exported to Reduce Visualize Gene Ontology (REViGO; http://revigo.irb.hr/) [37] for semantic representative subset analysis of nonredundant GO terms. GO terms were clustered based on their semantic similarity; in other words, similarly described biological functions of transcripts were grouped into a single classification, which facilitated simple visualization of the functional significance of groups of differentially expressed transcripts. GO enrichments were represented as scatterplots, in which the enriched terms were represented in a two-dimensional space derived by applying multidimensional scaling to a matrix of the GO terms’ semantic similarities. GO function analysis categorized the selected genes into groups based on three independent classification standards, namely molecular function (MF), cellular component (CC), and biological process (BP). The results were visualized in the form of three GO maps by using Cytoscape (version 3.4.0; http://cytoscape.org/). For visualization of genes and their associated pathways, the selected genes were accessed using WebGIVI (http://raven.anr.udel.edu/webgivi/) [38]. Pathways involving genes with significant differences and related gene regulation were obtained using online tool PathVisio [39] and DNA Intelligence Analysis (DIANA) miRPath (version 3.0) [40] from KEGG Pathways.

### 2.4. Flow Cytometry for Apoptosis and ROS Detection

For cell apoptosis analysis, astrocytes (5 × 10^5^ cells) were seeded and treated with IS (10 μM) for 24 and 48 h. Both floating cells and attached cells were harvested. Cell apoptosis was assessed using a BD Accuri C6 flow cytometry setup (BD Biosciences, Franklin Lakes, NJ, USA) by labeling annexin V-fluorescein isothiocyanate and propidium iodide apoptosis detection kit (BD Biosciences). For intracellular ROS analysis, astrocytes were exposed to control solution or IS for the specified time intervals (1, 2, and 3 h). Levels of intracellular H_2_O_2_ were measured using H_2_DCFDA (Molecular Probes, Waltham, MA, USA) fluorescent dye and determined using flow cytometry. Apoptosis referred to Annexin V positive and either propidium iodide positive or propidium iodide negative.

### 2.5. Mitochondrial Membrane Potential Assay

JC-1 dye is a mitochondrial membrane potential indicator. Astrocytes were seeded in a 96-well plate. Following treatment with IS for the specified time intervals (3, 6, 12, and 24 h), the cells were stained with 25 μM JC-1 (Invitrogen, Carlsbad, CA, USA) for 30 min at 37 °C. The samples were analyzed using a BD Accuri C6 flow cytometry setup [41]. JC-1 is a monomer when the membrane potential (ΔΨ) is lower than 120 mV, and it emits a green light (540 nm) following excitation by blue light (490 nm). At higher membrane potentials, JC-1 monomers convert to J-aggregates that emit a red light (590 nm) following excitation by green light (540 nm). Fluorescence was monitored using a fluorescence plate reader at wavelength pairs of 490 nm (excitation)/540 nm (emission) and 540 nm (excitation)/590 nm (emission). Changes in the ratio of fluorescence intensities corresponding to wavelengths of 590 nm (red) and 540 nm (green) were indicative of changes in the mitochondrial membrane potential.

### 2.6. Immunoblot Assay

Astrocytes were treated with IS (10 μM) for the specified time intervals (3, 6, 12, and 24 h), and the cells were lysed in radioimmunoprecipitation assay (RIPA) buffer (0.5 M Tris-HCl, pH 7.4, 1.5 M NaCl, 2.5% deoxycholic acid, 10% NP-40, 10 μM EDTA; Millipore Corporation, Billerica, MA, USA) containing a protease inhibitor cocktail (Sigma, St. Louis, MO, USA) on ice for 60 min. After the cell lysate was centrifuged at 4 °C and 12,000× *g* for 15 min, the supernatant fraction was collected for immunoblotting. The nuclear protein was extracted using a nuclear extract kit (Active Motif Europe, Rixensart, Belgium) according to the manufacturer’s instructions. Equivalent amounts of protein were resolved through sodium dodecyl sulfate–polyacrylamide gel electrophoresis (6%–12%) and transferred onto polyvinylidene difluoride membranes. After blocking for 1 h in 5% nonfat dry milk in Tris-buffered saline, the membrane was incubated with the desired primary antibody overnight at 4 °C, followed by peroxidase-conjugated secondary antibody for 1 h at room temperature. The applied antibodies were anti-phospho-ERK 1/2 (1:1000, cat. no.4370S, Cell Signaling), anti-ERK 1/2 (1:2000, cat. no.4695S, Cell Signaling), anti-phospho-c-Jun N-terminal kinase (JNK; 1:1000, cat. no.4668, Cell Signaling), anti-JNK (1:2000, cat. no.9258, Cell Signaling), anti-phospho-MAPK/ERK kinase (MEK; 1:1000, cat. no.9154, Cell Signaling), anti-MEK (1:2000, cat. no.8727, Cell Signaling), anti-phospho-p38 (1:1000, cat. no.9211, Cell Signaling), anti-p38 (1:2000, cat. no.8690, Cell Signaling), and anti-NRF-2 (1:2000, cat. no.12721, Cell Signaling). The protein bands were detected using an enhanced chemiluminescence kit (Millipore, Bedford, MA, USA) on an Alpha Innotech FluorChem FC2 imaging system (ProteinSimple; Bio-Techne, Minneapolis, MN, USA). Each membrane was divided or stripped to examine the levels of loading control (glyceraldehyde 3-phosphate dehydrogenase [GAPDH] or lamin A/C). Quantification was performed using ImageJ (version 1.51, National Institutes of Health, Bethesda, MD, USA).

### 2.7. Statistical Analysis

The gene expression levels were compared between the IS-treated astrocytes and the controls by performing nonparametric analysis based on the Mann–Whitney U test. All statistical analyses were performed using STATA (version 14; StataCorp LP, TX, USA) or GraphPad Prism (version 5; GraphPad Software, La Jolla, CA, USA). *p* < 0.05 (two-tailed) was considered to indicate a statistically significant between-group difference. Because of multiple testing for gene function or pathway enrichment analyses, a false discovery rate (FDR)-adjusted *p*-value of <0.05 was used.

## 3. Results

### 3.1. Effect of IS on Cell Viability and IS-Induced Cell Apoptosis in Human Astrocytes

**Figure 2:**
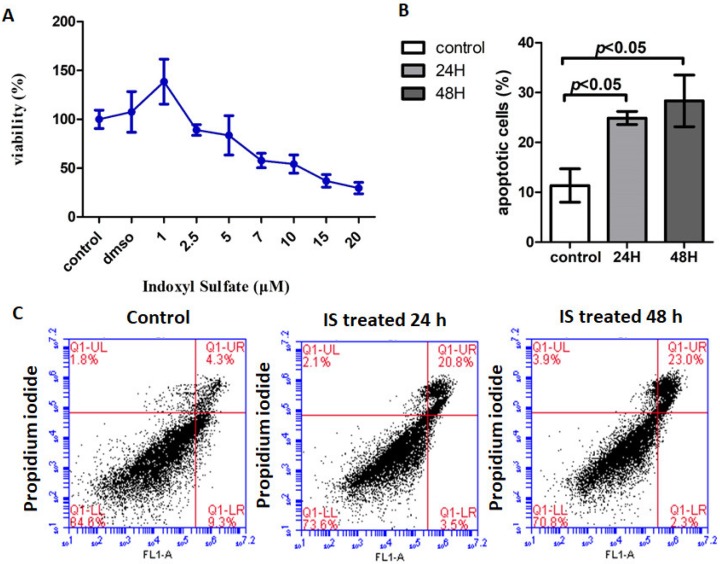
The cellular toxicity of IS treated human astrocyte. (**A**) Effect of IS on cell viability of human astrocytes. Cell viability was assessed with WST-1 assay after 72 h of treatment with different concentrations of IS. The cell toxicity of IS was found to be dose dependent. Exposure to 1, 2.5, 5, 7.5, 10, 15, and 20 μM IS decreased cell viability. The graph represents mean cell viability (%) ± standard deviation (SD) of three independent experiments. (**B**) IS induced human astrocyte apoptosis at 24 h and 48 h compared to control. (**C**) Human astrocytes were treated in either control or IS for 24 h and 48 h. The results were analyzed using a flow cytometer with fluorescein-isothiocyanate-conjugated Annexin V and propidium iodide stain. Increased apoptosis was noted in the astrocytes treated with IS for 24 h and 48 h.

The cytotoxic effects of IS on the viability of astrocytes were assessed using an WST-1 assay. The cell toxicity of IS was found to be dose dependent. The 50% inhibitory concentration of IS on astrocytes was 10 μM (Figure 2A). Further flow cytometry analysis showed that apoptosis of the IS-treated astrocytes increased dramatically at 48 h in contrast to that of the controls (Figure 2B,C). Next-generation sequencing (NGS) analysis was performed to better understand the signal transduction of apoptosis.

### 3.2. Differentially Expressed mRNAs between IS-Treated Astrocytes and Controls

**Figure 3:**
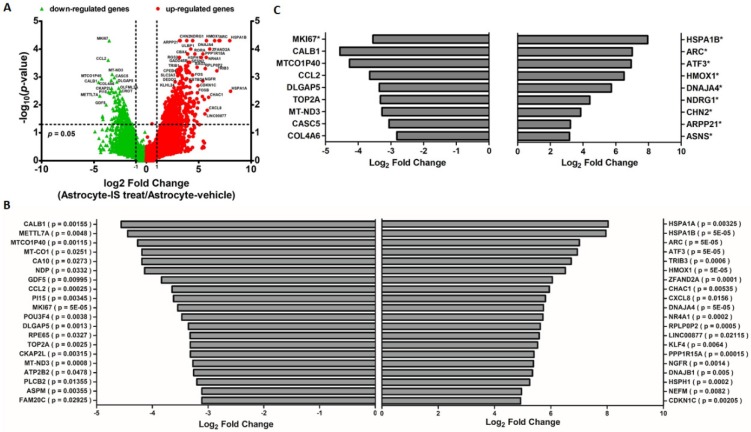
Display of differential expression patterns of IS-treated and vehicle-treated human astrocyte from deep sequencing. (**A**) Volcano plot of the RNA sequencing result of differential gene expression in IS and vehicle-treated astrocyte. The x-axis indicates the logarithm to the base 2 of expression fold-change (astrocyte-IS treat/Astrocyte-vehicle), and the y-axis indicates the negative logarithm to the base 10 of the *p*-values. Red circular marks represent upregulated genes in the Astrocyte-IS treat group, and green triangular marks represent downregulated genes in the Astrocyte-IS treat group. Vertical lines reflect the filtering thresholds of 2.0-fold-change, and horizontal line reflect filtering threshold of *p*-value = 0.05. A total of 437 significantly upregulated and 191 significantly downregulated genes in IS-treated astrocytes can be identified. (**B**) Bar graphs display the top 20 upregulated and 20 downregulated genes in IS-treated astrocytes with *p*-value < 0.05. (**C**) Bar graphs display the upregulated and downregulated genes in IS-treated astrocytes with a false discovery rate (FDR) adjusted *p*-value (* indicates FDR-adjusted *p*-value < 0.05).

RNAs were extracted from the astrocytes treated with 10 μM IS and sent for NGS followed by bioinformatics analyses. The volcano plot showed differentially expressed genes (DEGs) detected by RNA-seq in IS-treated and nontreated astrocytes (Figure 3A). The 20 most significant differences among the regulated genes are shown for both upregulated DEGs and downregulated DEGs (Figure 3B). The top regulated genes with FDR adjustment are listed in Figure 3C. In addition, the DEGs were analyzed with CC ontology, which describes the location of gene product in the cell (Figure S1A). The scatterplot generated using the REViGO visualization tools identified cytoplasm and nucleus as the major CCs of DEGs (Figure S1B).

### 3.3. Analysis of GO Terms Associated with DEGs

**Figure 4:**
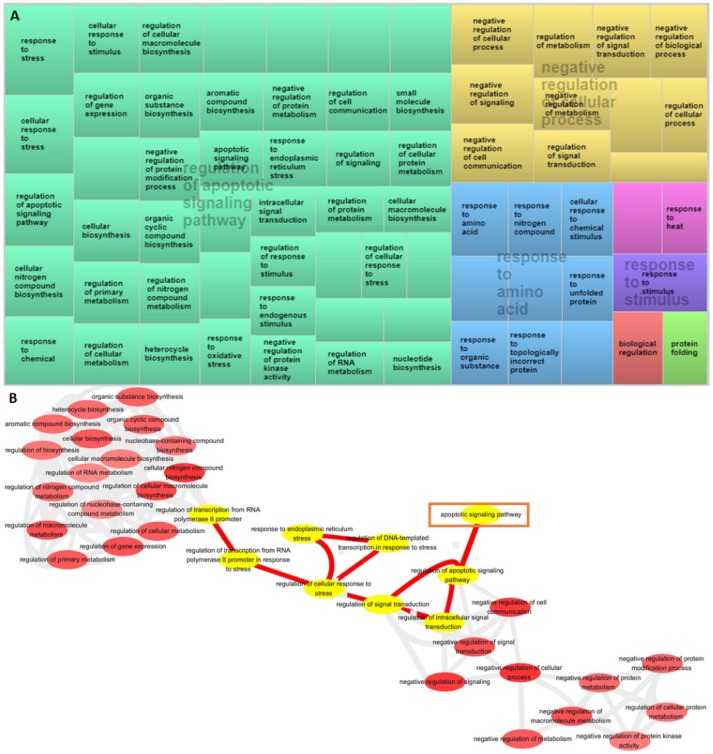
Gene Ontology (GO) enrichment analysis of differentially expressed genes based on over-representation analysis (ORA). (**A**) Enrichment of DEGs among biological process category in IS-treated astrocytes. Treemaps of DEGs generated using REVIGO. Each rectangle is a single cluster representative. The representatives are joined into “superclusters” of loosely related terms, visualized with different colors. Similar colors denote semantic similarity, and the dimension of the area is proportional to the overall direction of impact. Sizes of the rectangles reflect either the *p*-value or the frequency of the GO term in a given cluster. (**B**) REVIGO interactive graph accessed using Gorilla-summarized gene ontology biological process categories.

The BPs, CCs, and MFs identified using WebGestalt were all similar based on the over-representation analysis (ORA) or gene set enrichment analysis (GSEA) approach (Figure S2A). The results of GO analysis of each set of DEGs from the three workflows—DAVID, Gorilla, and CateGOrizer—were consistent. Enriched method ranked by combined score (*p*-value multiplied by z-score) showed the top regulated BP as a response to unfolded protein, positive regulation of transcription from RNA polymerase II promoter, and regulation of apoptosis process (Figure S2B). Enriched categories obtained using the Gorilla algorithm implicated the crucial roles of regulation of apoptotic signaling pathway and negative regulation of cellular process (Figure 4A) in BPs. Visualization of the BPs and differences between the RNA fractions were performed using the CateGOrizer algorithm and REViGO semantic similarity-based scatterplots. Based on the above findings, the summarized BPs of DEGs were related to protein folding, biological regulation, regulation of cellular response to stress, and regulation of apoptotic signaling pathway (Figure S2C). The BP interactive graph obtained using the Gorilla algorithm and from the REViGO analyses identified the relationship among cellular response to stress, signal transduction, and regulation of apoptotic signaling pathway (Figure 4B). Details of the GO BP are listed in Table S2.

### 3.4. Analysis of Pathway Enrichment with DEGs

**Figure 5:**
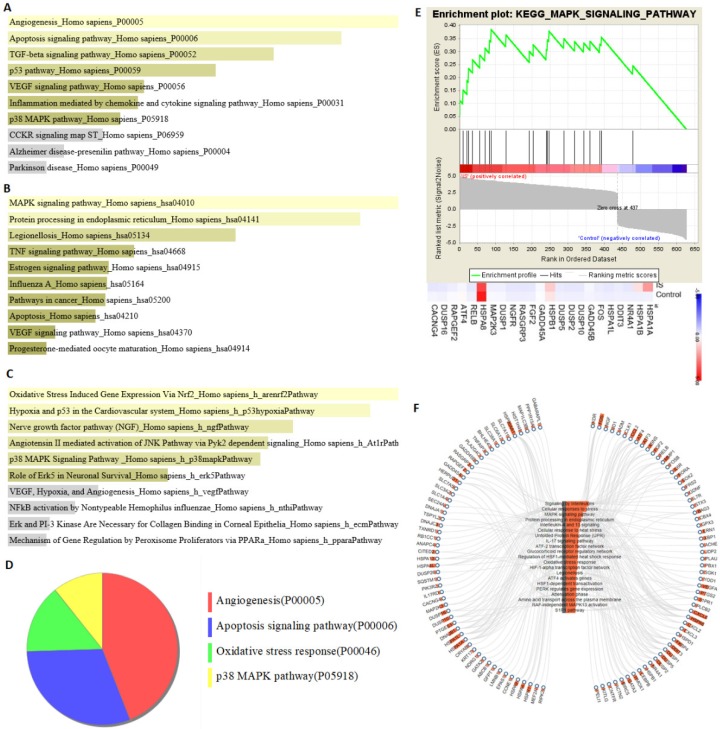
Pathway enrichment analysis of differentially expressed genes. The results of (**A**) PANTHER (Protein ANalysis THrough Evolutionary Relationships), (**B**) KEGG (Kyoto Encyclopedia of Genes and Genomes), and (**C**) BioCarta pathway enrichment analyses performed using Enrichr method. (**D**) The major PANTHER pathway identified using the Benjamini–Hochberg false discovery rate (FDR) multiple test correction. (**E**) The Gene Set Enrichment Analysis (GSEA) result of differentially expressed genes. The 628 differentially expressed genes in IS-treated astrocytes were uploaded into GSEA for enrichment analysis. The KEGG gene sets database was used as the gene set collection for analysis. GSEA performed 1000 permutations. The cutoff for significant gene sets was a false discovery rate <25%. (**F**) The correlation of KEGG pathway and associated gene expression on IS-treated astrocytes using WebGIVI web-based gene visualization tool. The bar chart on the node represents the frequency of the said node.

Pathway enrichment analysis was performed using the Enrichr method. PANTHER enrichment indicated that the apoptosis signaling pathway and the p38 MAPK pathway were involved in the pathophysiology of astrocytes treated with IS (Figure 5A). Moreover, KEGG enrichment indicated the MAPK signaling pathway, protein processing in endoplasmic reticulum, and apoptosis pathway (Figure 5B). Moreover, the BioCarta pathway enrichment suggested stimulation of oxidative stress and the p38 MAPK pathway (Figure 5C). PANTHER with FDR multiple test correction demonstrated oxidative stress response, p38 MAPK, and apoptosis signaling as the key pathways (Figure 5D). Further enrichment analysis with FDR adjustment by using GSEA for the KEGG database demonstrated MAPK as the key pathway. The heat maps of the potentially involved genes in treated/nontreated IS astrocytes were *HSPA1A*, *HSPB1*, and *HSPA8* (Figure 5E). The correlation of the KEGG pathway and the associated gene expression in the case of IS-treated astrocytes was shown using the WebGIVI visualization tool (Figure 5F). The top KEGG and PANTHER enrichment pathways and the related genes determined using the ORA or the GESA approach are listed in Tables S3 and S4.

### 3.5. IS-Triggered Astrocyte Toxicity and Associated Regulating Pathways

**Figure 6:**
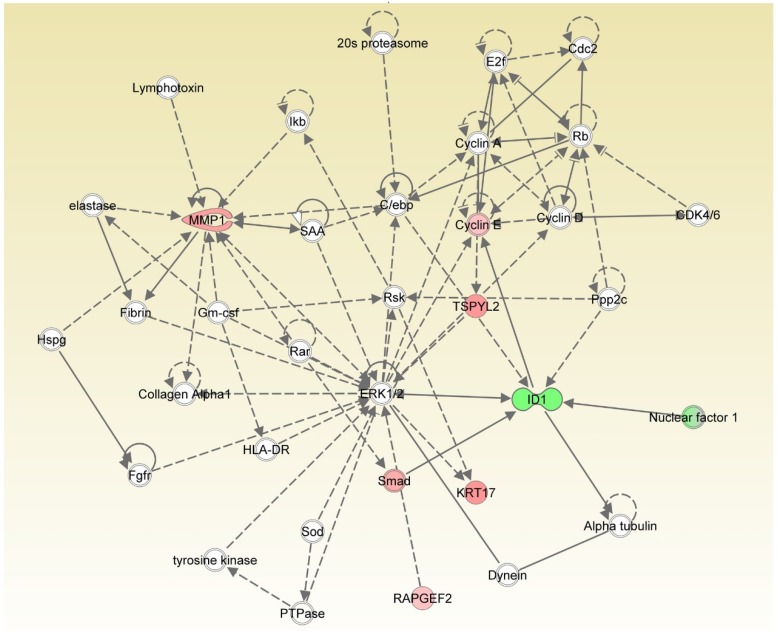
Network analysis of differentially expressed genes. Central gene identification according to IPA core analysis. ERK is the central gene in the interaction network.

To identify the pathogenesis of the effect of IS on astrocytes, we performed pathway enrichment analyses by using CPDB to combine the results of multiple databases (KEGG, Wiki Pathways, Reactome, and BioCarta) and identify relevant pathways. Oxidative stress, NRF-2, MAPK signaling, and protein processing in endoplasmic reticulum were found to be the key pathways related to cell apoptosis (Figure S3). The shared selected DEGs in IS-treated astrocytes corresponded to the regulated genes (HSPA1B and HMOX1). Interaction network of the central gene identified in the current study was the ERK pathway according to IPA core analysis (Figure 6). The MAPK signaling pathway from KEGG (ID: hsa04010) overlaid with log_2_ fold change values obtained using PathVisio indicated upregulation of MAPK phosphatases (MKP) in IS-treated astrocytes (Figure S4A). Dual-specificity phosphatase (DUSP) was found to be the key regulator on MKP among MAPK signal transductions by using DIANA mirPath (Figure S4B). Thus, IS-induced apoptosis of astrocytes occurred via the oxidative stress, NRF-2, and MAPK pathways. One of the key regulatory molecules was DUSP, which reduced the phosphorylation of ERK signaling by affecting MKP. An experiment was then performed to confirm the bioinformatics findings.

### 3.6. IS-Induced Astrocyte Apoptosis via ROS-NRF-2 Signaling Pathway

**Figure 7:**
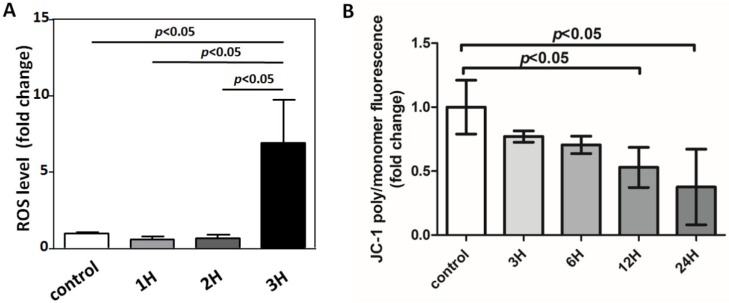
IS induced mitochondrial dysfunction and ROS production. (**A**) Cells treated with IS for various durations (1 h, 2 h, and 3 h), and ROS level determined by staining with H_2_DCFDA fluorescent dye, followed by flow cytometry analysis. The ROS increases markedly at 3 h after IS treatment. (**B**) IS-treated astrocytes induce loss of mitochondrial membrane potential as measured by JC-1 and flow cytometry at 12 and 24 h. Data represent mean ± SD of a representative experiment.

**Figure 8:**
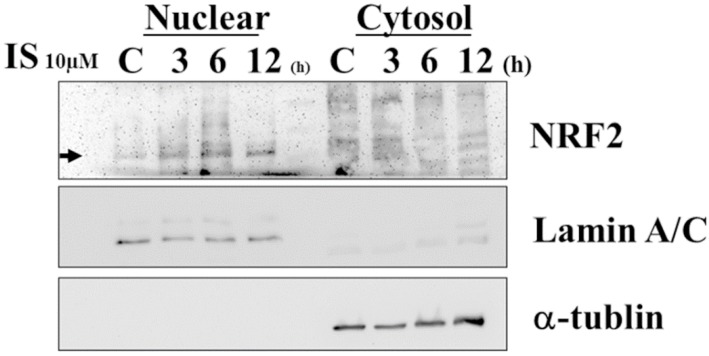
Representative immunoblot analysis of NRF2 in nucleus and cytosol. NRF2 was detected and normalized to lamin A/C in the nucleus and α-tubulin in cytosol. After astrocytes were treated with 10 μM IS for different durations (3, 6, and 12 h), NRF2 was examined in nuclear and cytosolic fractions. NRF2 protein expression was decreased in cytosol and increased in the nucleus.

Mitochondrial dysfunction, including aberrant ROS production and membrane potential, has been considered a critical mechanism for apoptotic cell death [42]. Therefore, we measured mitochondrial membrane potential by using the mitochondria-specific dye JC-1. IS enhances mitochondrial ROS production, along with a loss of mitochondrial membrane potential, as determined by JC-1 disaggregation at 12 and 24 h (Figure 7). To examine the effect of IS on the modulation of NRF2 nuclear translocation, human astrocytes were treated with IS for various periods. A decreased cytosolic NRF2 protein level and an increased nuclear NRF2 protein level were observed in IS-treated astrocytes (Figure 8).

### 3.7. IS-Inhibited MAPK Pathway with Regulation by DUSP in Astrocytes

**Figure 9:**
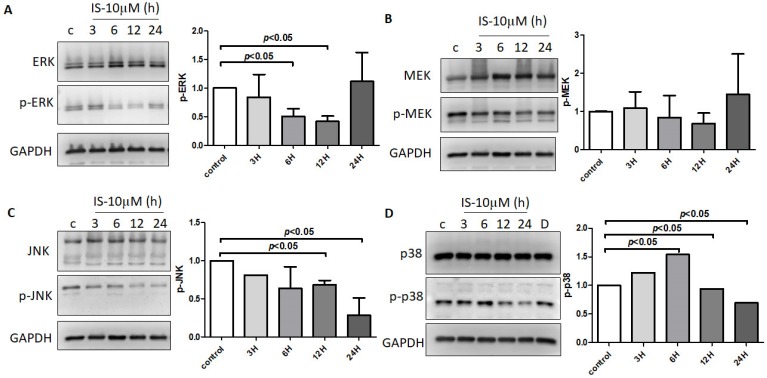
Human astrocytes treated with control or 10 μM IS for various durations (3 h, 6 h, 12 h, and 24 h). The phosphorylated and total protein levels in the cell lysates were assessed with an immunoblot assay. The results shown are representative of three independent experiments performed on different days, along with relative expression levels to the corresponding control groups at the same time point. IS decreased the phosphorylation of (**A**) ERK, (**B**) MEK, (**C**) JNK, and (**D**) p-38 at 12 h treatment in human astrocytes. Data represent mean ± SD of a representative experiment.

The effect of IS on MAPK signaling was assessed, and the phosphorylation of ERK, MEK, JNK, and p38 in human astrocytes for different durations was investigated. IS reduced the phosphorylation of several proteins on the MAPK pathway, such as ERK, MEK, JNK, and p-38 (Figure 9A–D). DUSPs (MKP) was identified as the key element in the MAPK pathway in bioinformatics enrichment analysis. The NGS analysis revealed that DUSP1, DUSP5, DUSP5, and DUSP16 were upregulated on astrocytes under IS treatment with fold change greater than 2 and FPKM greater than 5 (Table 1).

## 4. Discussion

**Figure 10:**
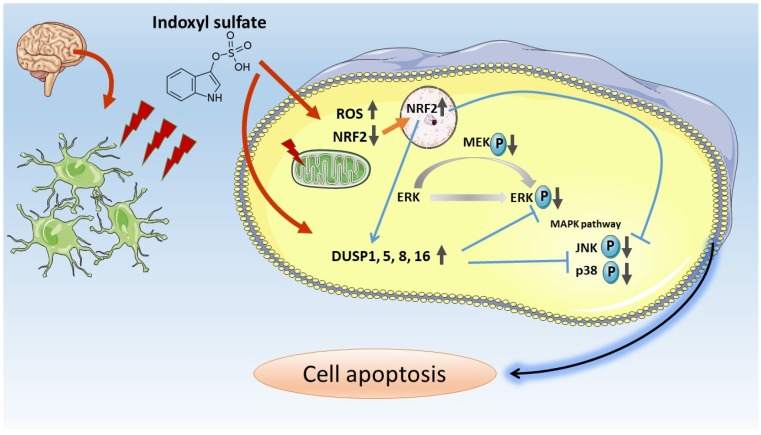
Scheme of proposed IS-induced apoptosis via ROS-NRF2 and MAPK signaling pathways in human astrocytes.

In the present study, we applied NGS analysis to IS-treated and control human astrocytes to explore the potential molecular mechanisms by employing bioinformatics approaches. We performed experiments to confirm the bioinformatics findings. Merged network analysis (KEGG, BioCarta, Wiki, and Reactome pathways) performed using ConsensusPathDB on candidate genes and pathways indicated that the NRF-2 pathway, MAPK signaling pathway, and apoptosis pathway were the main pathophysiological processes in IS-treated astrocytes. The pathway analysis of DEGs showed that the apoptosis, oxidative, and MAPK signaling pathways were the main signaling transductions in PANTHER, KEGG, and BioCarta pathway enrichment analyses, respectively (Figure 5). Moreover, the apoptosis signaling pathway in KEGG visualized with Pathvisio and DIANA miRPath revealed that ERK and DUSPs are the key molecules involved in the MAPK signaling pathway (Figure S4). *HSPA1B* and *HMOX1* were identified as the top dysregulated genes involved in ROS-NRF2 and MAPK signaling–associated cell apoptosis through systematic bioinformatics analysis (Table S2). Based on the NGS analysis and bioinformatics findings, we performed further immunoblotting to confirm IS regulation in the NRF2 pathway and the MAPK pathway in human astrocytes. Taken together, our results revealed that IS increased intracellular ROS levels and reduced mitochondrial membrane potential. Further investigation indicated that IS-induced astrocyte apoptosis via the nuclear translocation of NRF2. Moreover, IS downregulated proteins contributing to cell apoptosis along the MAPK pathway, such as ERK, MEK, JUK, and p38, through the effect of DUSPs. The proposed mechanism is shown in Figure 10.

IS could downregulate NRF2 expression through NF-κB activation, followed by hemeoxygenase-1 and NAD(P)H:quinone oxidoreductase 1 downregulation, thereby increasing ROS production [43]. ROS production induced by IS has been observed in several cells, such as renal tubular cells [43], endothelial cells [44,45,46,47,48], vascular smooth muscle cells, red blood cells [49], and monocyte and macrophages [50]. Reported in C6 glioma cells (astrocyte-like cell lines) and primary astrocytes with mixed glial cells, IS-induced activation of NF-kB, ROS, and pro-inflammatory cytokine production, and downregulation of cell-protective factors such as NRF-2, HO-1, or NQO1 [16]. Moreover, NRF-2 could downregulate p38, JNK, and ERK along the MAPK pathway by activating DUSPs [51,52,53]. In the present study, apoptosis was the most predominant expression in cell death. IS provoked ROS production; reduced NRF2 levels; increased DUSPs levels; downregulated ERK, JNK, and p38; and subsequently, triggered the MAPK apoptosis pathway in human astrocytes [54,55]. The MAPK pathway, which determines cell fate, including cell survival and death [56], was one of the key pathways found in the bioinformatics analysis.

The NGS analysis revealed that DUSP 1, 5, 8, and 16 were the most preeminent expressed genes in astrocytes treated with IS. These DUSPs could deactivate ERK by phosphatases and promote apoptosis simultaneously [57,58,59,60]. DUSPs expression and activity were regulated tightly at different levels, and they were found to finally depend on cell type and specific cellular context. DUSPs are inducible phosphatases and display broad specificity for inactivation of bisphosphorylated ERK, p38, and JNK MAP kinase families [61,62]. DUSP1 can switch off MAPK signal transduction via ERK, JNK, and p38 dephosphorylation [57,63]. DUSP5 is an inducible nuclear variant that specifically regulates the nuclear dephosphorylation and accumulation of ERK [58]. DUSP8 is a MAPK phosphatase that can dephosphorylate JNK and p38 [59,64]. DUSP16 preferentially deactivates JNK and p38 in the MAPK pathway [65].

In this study, we showed that dual-specificity phosphatases (DUSP) indeed regulate the MAPK signaling pathway by targeting ERK phosphorylation.

## 5. Conclusions

Based on the results of NGS with bioinformatics analysis and that of an experimental study on human astrocytes, we found that IS induced activation of ROS and downregulated cell-protective factors such as NRF2. Moreover, IS promoted cell apoptosis through MAPK signaling by reducing the phosphorylation of ERK, MEK, JNK, and p38 with DUSP 1, 5, 8, and 16. The results highlighted the neurotoxicity of IS on human astrocytes. Thus, the study provides partial evidence that protein-bound uremic toxins contribute to neurological complications observed in CKD. Lowering IS levels can potentially prevent cognitive dysfunction in patients with CKD.

## Figures and Tables

**Table 1:** Dual-specificity phosphatase (DUSP) mRNA expression measured by NGS analysis. Under the condition of fragments per kilobase of transcript per million (FPKM) >5 and fold change >2. IS increased the expression of DUSP1, DUSP5, DUSP5, and DUSP16 in astrocytes.

Gene ID	Associated Gene Name	EntrezGene ID	Locus	FPKM (NHA-IS-10μM)	FPKM (NHA)	Ratio Ratio (NHA-IS-10μM/NHA)
ENSG00000079393	DUSP13	51207	10:75094431-75182123	0.219633	0.0001	2196.33
ENSG00000111266	DUSP16	80824	12:12474209-12562383	52.219	14.1532	3.689554
ENSG00000120129	DUSP1	1843	5:172758225-172777774	221.988	38.2398	5.805156
ENSG00000130829	DUSP9	1852	X:153642491-153651326	0.169448	0.070583	2.400708
ENSG00000133878	DUSP26	78986	8:33591331-33600106	3.46896	0.421005	8.239712
ENSG00000138166	DUSP5	1847	10:110497837-110511544	127.022	9.39548	13.51948
ENSG00000143507	DUSP10	11221	1:221701423-221742176	25.8229	1.7915	14.41412
ENSG00000149599	DUSP15	128853	20:31847636-31952092	27.5482	4.7307	5.823282
ENSG00000158050	DUSP2	1844	2:96143165-96145440	38.0541	2.74733	13.8513
ENSG00000162999	DUSP19	142679	2:183078558-183108519	0.118331	0.633702	0.18673
ENSG00000167065	DUSP18	150290	22:30635651-30669016	3.83218	9.21196	0.416001
ENSG00000184545	DUSP8	1850	11:1554043-1599184	36.2799	6.57598	5.517033
ENSG00000189037	DUSP21	63904	X:44844003-44844888	0.147912	0.0001	1479.12

## Supplementary Materials

The following are available online at http://www.mdpi.com/2077-0383/8/2/191/s1, Figure S1: Differentially regulated genes in IS-treated astrocytes classified according to protein class and cellular components, Figure S2: Gene ontology (GO) enrichment analysis of differentially expressed genes in IS-treated astrocytes, Figure S3: Network analysis of candidate gene and pathway correlated to IS-induced apoptosis signal, Figure S4: KEGG Apoptosis signaling pathway in IS-treated astrocytes, Table S1: Top 10 upregulated and downregulated genes in indoxyl sulfate-treated human astrocytes, Table S2: Gene Ontology (GO) biological process enrichment analysis of differentially expressed genes, Table S3: Top 10 KEGG and PANTHER pathway enrichment analysis of differentially expressed genes based on overrepresentation enrichment analysis using WebGestalt, Table S4: Top 10 KEGG and PANTHER pathway enrichment analysis of differentially expressed genes based on gene set enrichment analysis using WebGestalt.

## Abbreviations

IS	indoxyl sulfate
CKD	chronic kidney disease
MAPK	mitogen-activated protein kinase
