# Supplementary material for: Indoxyl Sulfate Induces Apoptosis through Oxidative Stress and Mitogen-Activated Protein Kinase Signaling Pathway Inhibition in Human Astrocytes
Source: J Clin Med. 2019 Feb 5;8(2):191. doi: 10.3390/jcm8020191 (PMC6406290; doi:10.3390/jcm8020191)
Supplement: Supplementary file 1 [file jcm-08-00191-s001.pdf]

Supplementary Figure 1

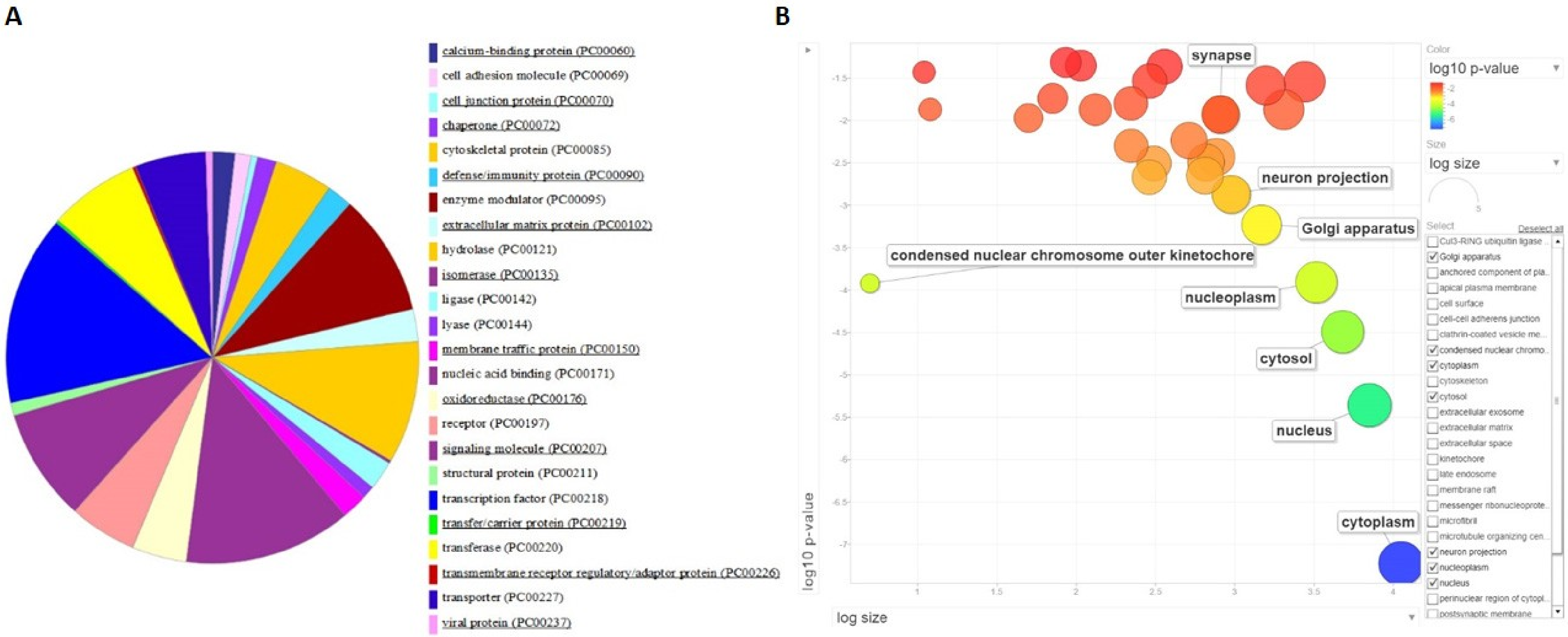

Figure S1. (**A**) Differentially regulated genes in IS-treated astrocytes were classified according to the protein class categorized by PANTHER (Protein ANalysis THrough Evolutionary Relationships) protein class. (**B**) Scatterplot of gene product cellular components generated from differentially expressed genes using REViGO visualizations tools. GO terms are represented by circles and are plotted according to log size on the x-axis and $\log_{10}$ *p*-value on the y-axis. The size is proportional to frequency of the GO term. The color of the circle defines the $\log_{10}$ *p*-value (red is larger, blue is smaller).

Figure S2

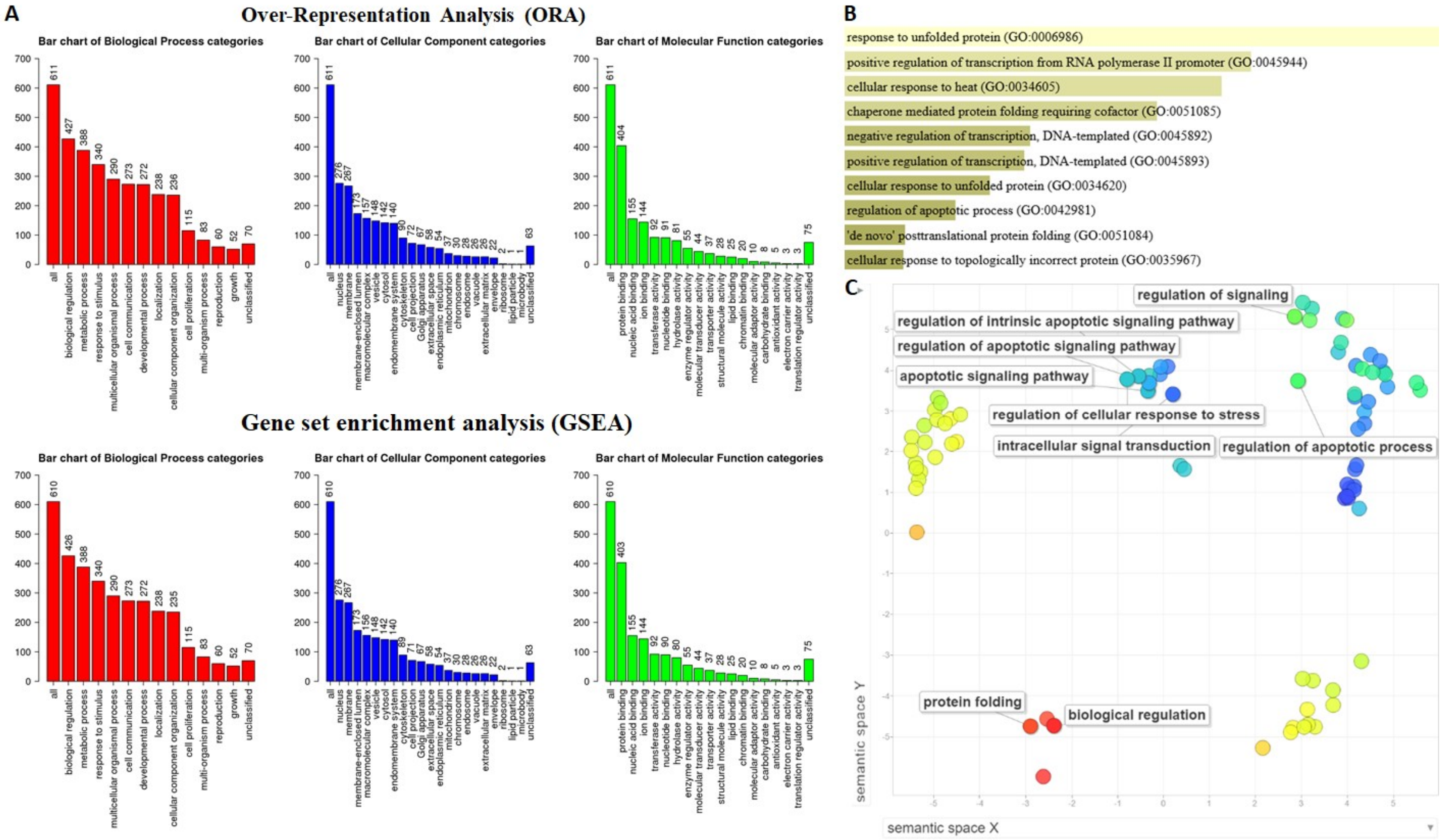
A
Over-Representation Analysis (ORA)
Bar chart of Biological Process categories
Bar chart of Cellular Component categories
Bar chart of Molecular Function categories
Gene set enrichment analysis (GSEA)
Bar chart of Biological Process categories
Bar chart of Cellular Component categories
Bar chart of Molecular Function categories
B
response to unfolded protein (GO:0006986)
positive regulation of transcription from RNA polymerase II promoter (GO:0045944)
cellular response to heat (GO:0034605)
chaperone mediated protein folding requiring cofactor (GO:0051085)
negative regulation of transcription, DNA-templated (GO:0045892)
positive regulation of transcription, DNA-templated (GO:0045893)
cellular response to unfolded protein (GO:0034620)
regulation of apoptotic process (GO:0042981)
'de novo' posttranslational protein folding (GO:0051084)
cellular response to topologically incorrect protein (GO:0035967)
C
regulation of signaling
regulation of intrinsic apoptotic signaling pathway
regulation of apoptotic signaling pathway
apoptotic signaling pathway
regulation of cellular response to stress
intracellular signal transduction
regulation of apoptotic process
protein folding
biological regulation
semantic space Y
semantic space X

Figure S2. (**A**) Gene ontology (GO) enrichment analysis of differentially expressed genes based on over-representation analysis (ORA) or gene set enrichment analysis (GSEA). The identified genes were analyzed according to GO enrichment using WebGestalt (WEB-based GEne SeT AnaLysis Toolkit). GO classification included the biological process and molecular function ontology enrichment. Biological process, cellular component, and molecular function categories are represented by red, blue, and green bars, respectively. The heights of the bars represent the numbers of user list genes observed in the category. (**B**) GO biological process ranked by combined score (*p*-value multiplied by z-score) associated with genes modulated using Enrichr analysis tool. The top figure depicts a bar graph from the top 10 upregulated GO biological processes. (**C**) The biological processes were classified into broad groups based on the GO-slim classification system by using CateGOrizer and visualized as REViGO semantic similarity-based scatterplots. Individual circles indicate cluster representatives (terms remaining after reducing redundancy) in a two-dimensional space derived by applying multi-dimensional scaling to a matrix of GO terms with semantic similarities. Bubble color indicates the *p*-value (legend in upper right-hand corner); the two ends of the colors are red and blue, depicting lower- and higher *p*-values, respectively. Size indicates the relative frequency of the GO term in the underlying reference European Bioinformatics Institute Gene Ontology Annotation (EBI-GOA) database. More functionally similar GO terms are closer in the scatterplot.

Figure S3

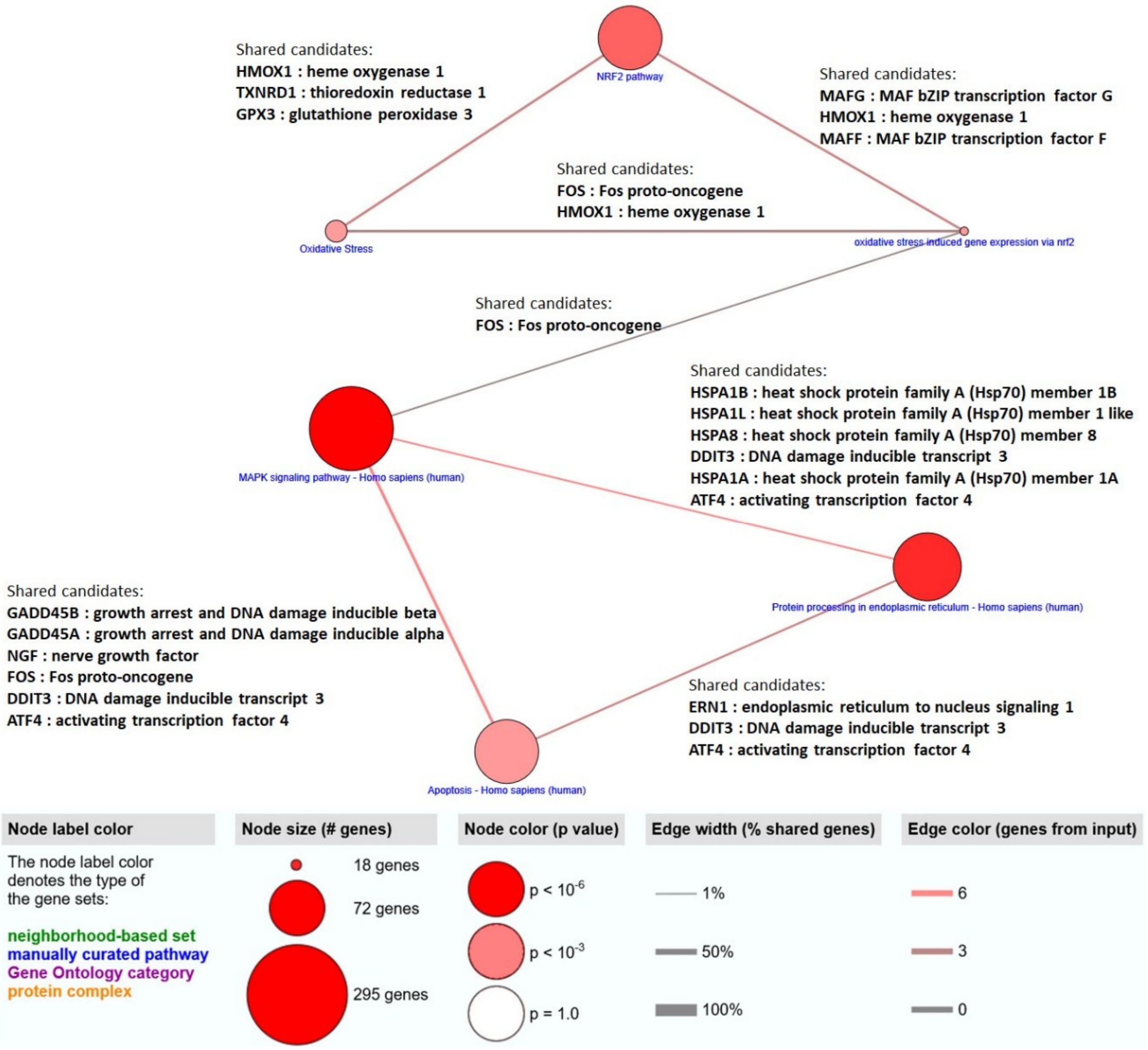

Figure S3. Merged network analysis (KEGG, Biocarta, Wiki, and Reactome pathways) from ConsensusPathDB for candidate gene and pathway correlated to IS-induced apoptosis signal.

Figure S4

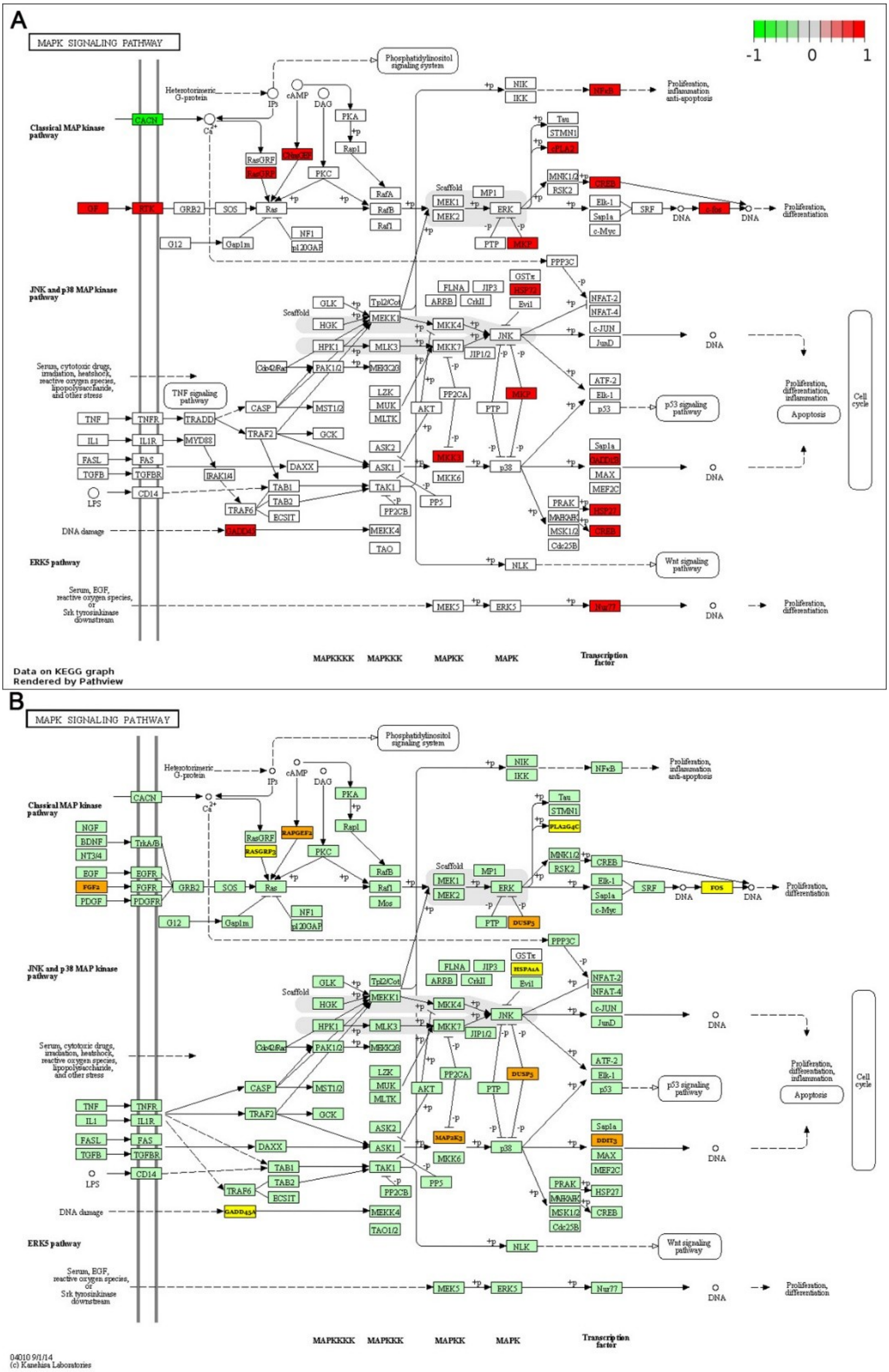

A
MAPK SIGNALING PATHWAY
-1
0
1
Phosphatidylinositol signaling system
Heterotrimeric G-protein
IP3
cAMP
DAG
Ca2+
Classical MAP kinase pathway
CACN
GF
RTK
GRB2
SOS
Ras
RasGRF
CNrasGEF
PKC
PKA
Rap1
RafA
RafB
Raf1
NF1
p120GAP
G12
Gap1m
Scaffold
MP1
MEK1
MEK2
ERK
PTP
MKP
NIK
IKK
NFκB
Proliferation, inflammation, anti-apoptosis
Tau
STMN1
cPLA2
MNK1/2
RSK2
CREB
Elk-1
Sap1a
c-Myc
SRF
DNA
c-fos
Proliferation, differentiation
PPP3C
JNK and p38 MAP kinase pathway
GSTπ
HSP72
FLNA
JIP3
ARRB
CrkII
Evi1
NFAT-2
NFAT-4
c-JUN
JunD
GLK
HGK
Tpl2/Cot
MEKK1
MKK4
JNK
HPK1
MLK3
MKK7
JIP1/2
Cdc42/Rac
PAK1/2
MEKK2/3
Serum, cytotoxic drugs, irradiation, heatshock, reactive oxygen species, lipopolysaccharide, and other stress
TNF signaling pathway
CASP
MST1/2
LZK
MUK
MLTK
AKT
PP2CA
ATF-2
Elk-1
p53
p53 signaling pathway
Proliferation, differentiation, inflammation
Apoptosis
Cell cycle
TNF
TNFR
TRADD
IL1
IL1R
MYD88
TRAF2
GCK
FASL
FAS
DAXX
TGFB
TGFBR
IRAK1/4
LPS
CD14
ASK2
ASK1
MKK3
MKK6
p38
TAB1
TAB2
TAK1
TRAF6
ECSIT
PP2CB
PP5
Sap1a
GADD153
MAX
MEF2C
PRAK
MAPKAPK
MSK1/2
Cdc25B
HSP27
CREB
DNA damage
GADD45
MEKK4
TAO
NLK
Wnt signaling pathway
ERK5 pathway
Serum, EGF, reactive oxygen species, or Srk tyrosinkinase downstream
MEK5
ERK5
Nur77
MAPKKKK
MAPKKK
MAPKK
MAPK
Transcription factor
Data on KEGG graph
Rendered by Pathview
B
MAPK SIGNALING PATHWAY
Phosphatidylinositol signaling system
Classical MAP kinase pathway
CACN
NGF
BDNF
NT3/4
TrkA/B
EGF
EGFR
FGF2
FGFR
PDGF
PDGFR
GRB2
SOS
Ras
RasGRF
RAPGEF2
RASGRP3
PKC
PKA
Rap1
RafB
Raf1
Mos
NF1
p120GAP
G12
Gap1m
MEK1
MEK2
MP1
ERK
PTP
DUSP5
NIK
IKK
NFκB
Tau
STMN1
PLA2G4C
MNK1/2
RSK2
CREB
Elk-1
Sap1a
c-Myc
SRF
FOS
PPP3C
JNK and p38 MAP kinase pathway
GSTπ
HSPA1A
Evi1
NFAT-2
NFAT-4
c-JUN
JunD
MKK4
MKK7
JNK
DUSP5
PP2CA
MAP2K3
p38
DDIT3
GADD45A
MEKK4
TAO1/2
NLK
HSP27
CREB
MEK5
ERK5
Nur77
MAPKKKK
MAPKKK
MAPKK
MAPK
Transcription factor
04010 9/1/14
(c) Kanehisa Laboratories

Figure S4. Apoptosis signaling pathway in KEGG visualized with Pathvisio and DIANA-miRPath. (**A**) MAPK signaling pathway from KEGG (ID: hsa04630) overlaid with log_2 fold change values using PathVisio indicating up (red) or downregulation (blue) in IS-treated astrocytes. The scale for log_2 fold change values is indicated at the bottom of the pathway diagram. Genes not significantly differentially expressed are depicted in grey. (**B**) Regulatory factors are indicated with orange and yellow color, where yellow denotes gene targeted by 1 selected miRNA, and orange denotes gene targeted by >1 selected miRNAs.

Table S1. Top 10 upregulated and downregulated genes in indoxyl sulfate-treated human astrocytes.

Gene Symbol	Gene Name	IS-Treated Astrocyte FPKM	Control Astrocyte FPKM	Fold-Change(IS/Control)
HSPA1A	heat shock protein family A (Hsp70) member 1A	2966.26	11.3338	261.7180469
HSPA1B	heat shock protein family A (Hsp70) member 1B	1282.33	5.16669	248.1917824
ARC	activity-regulated cytoskeleton-associated protein	122.404	0.947772	129.1492047
ATF3	activating transcription factor 3	798.302	6.50267	122.7652641
TRIB3	tribbles pseudokinase 3	252.607	2.38762	105.7986614
HMOX1	heme oxygenase 1	1127.7	12.3371	91.40721888
ZFAND2A	zinc finger, AN1-type domain 2A	831.285	12.5355	66.31446691
CHAC1	ChaC glutathione-specific gamma-glutamylcyclotransferase 1	94.319	1.53066	61.61982413
CXCL8	chemokine (C-X-C motif) ligand 8	54.3667	0.9702	56.03659039
DNAJA4	DnaJ heat shock protein family (Hsp40) member A4	446.764	8.3832	53.29277603
CALB1	calbindin 1	2.33229	55.0017	0.042403962
METTL7A	methyltransferase like 7A	0.41223	8.9641	0.045986769
MTCO1P40	MT-CO1 pseudogene 40	11.4618	220.025	0.052093171
MT-CO1	mitochondrially encoded cytochrome c oxidase I	317.248	5778.42	0.054902205
CA10	carbonic anhydrase X	0.391177	7.12425	0.054907815
NDP	Norrie disease (pseudoglioma)	1.5142	26.6683	0.056779022
GDF5	growth differentiation factor 5	0.845582	12.0735	0.070036195
CCL2	chemokine (C-C motif) ligand 2	70.9431	888.045	0.07988683
PI15	peptidase inhibitor 15	0.471967	5.8017	0.081349777
MKI67	marker of proliferation Ki-67	3.14015	36.6556	0.08566631

Table S2. Gene Ontology (GO) biological process enrichment analysis of differentially expressed genes.

GO Term	Description	*p*-Value	FDR *q*-Value
GO:0006950	response to stress	2.79×10^{-7}	1.73×10^{-3}
GO:0050896	response to stimulus	2.05×10^{-6}	4.23×10^{-3}
GO:0043067	regulation of programmed cell death	9.02×10^{-6}	5.57×10^{-3}
GO:0023057	negative regulation of signaling	8.26×10^{-6}	5.67×10^{-3}
GO:0010941	regulation of cell death	1.23×10^{-5}	5.86×10^{-3}
GO:2001234	negative regulation of apoptotic signaling pathway	1.05×10^{-5}	5.88×10^{-3}
GO:0033554	cellular response to stress	1.94×10^{-6}	5.99×10^{-3}
GO:0042221	response to chemical	1.22×10^{-5}	6.26×10^{-3}
GO:0010648	negative regulation of cell communication	8.26×10^{-6}	6.38×10^{-3}
GO:0051716	cellular response to stimulus	1.73×10^{-5}	6.70×10^{-3}
GO:0042981	regulation of apoptotic process	7.66×10^{-6}	6.77×10^{-3}
GO:2000060	positive regulation of ubiquitin-dependent protein catabolic process	1.68×10^{-5}	6.92×10^{-3}
GO:0032436	positive regulation of proteasomal ubiquitin-dependent protein catabolic process	1.68×10^{-5}	7.41×10^{-3}
GO:0044271	cellular nitrogen compound biosynthetic process	7.21×10^{-6}	7.43×10^{-3}
GO:2000112	regulation of cellular macromolecule biosynthetic process	2.15×10^{-5}	7.82×10^{-3}
GO:1903052	positive regulation of proteolysis involved in cellular protein catabolic process	4.21×10^{-5}	8.13×10^{-3}
GO:0048523	negative regulation of cellular process	6.60×10^{-6}	8.16×10^{-3}
GO:1901800	positive regulation of proteasomal protein catabolic process	4.21×10^{-5}	8.39×10^{-3}
GO:2000058	regulation of ubiquitin-dependent protein catabolic process	4.21×10^{-5}	8.67×10^{-3}
GO:2001243	negative regulation of intrinsic apoptotic signaling pathway	3.84×10^{-5}	8.79×10^{-3}
GO:0048519	negative regulation of biological process	3.99×10^{-5}	8.80×10^{-3}
GO:0032434	regulation of proteasomal ubiquitin-dependent protein catabolic process	4.21×10^{-5}	8.97×10^{-3}

GO:0051172	negative regulation of nitrogen compound metabolic process	3.79×10^{-5}	9.01×10^{-3}
GO:2001233	regulation of apoptotic signaling pathway	5.88×10^{-6}	9.08×10^{-3}
GO:0043200	response to amino acid	3.71×10^{-5}	9.18×10^{-3}
GO:0080090	regulation of primary metabolic process	5.36×10^{-5}	9.46×10^{-3}
GO:0031324	negative regulation of cellular metabolic process	5.24×10^{-5}	9.53×10^{-3}
GO:0060255	regulation of macromolecule metabolic process	3.71×10^{-5}	9.55×10^{-3}
GO:0044249	cellular biosynthetic process	5.13×10^{-5}	9.61×10^{-3}
GO:0032269	negative regulation of cellular protein metabolic process	6.15×10^{-5}	9.74×10^{-3}
GO:0019222	regulation of metabolic process	2.85×10^{-5}	9.78×10^{-3}
GO:0009966	regulation of signal transduction	6.37×10^{-5}	9.84×10^{-3}
GO:0010468	regulation of gene expression	3.67×10^{-5}	9.85×10^{-3}
GO:0071495	cellular response to endogenous stimulus	6.55×10^{-5}	9.88×10^{-3}
GO:0009892	negative regulation of metabolic process	5.76×10^{-5}	9.89×10^{-3}
GO:0031323	regulation of cellular metabolic process	5.92×10^{-5}	9.89×10^{-3}
GO:2001242	regulation of intrinsic apoptotic signaling pathway	6.14×10^{-5}	9.99×10^{-3}
GO:0043618	regulation of transcription from RNA polymerase II promoter in response to stress	3.27×10^{-5}	1.01×10^{-2}
GO:0010605	negative regulation of macromolecule metabolic process	3.43×10^{-5}	1.01×10^{-2}
GO:0009968	negative regulation of signal transduction	3.59×10^{-5}	1.01×10^{-2}
GO:0043620	regulation of DNA-templated transcription in response to stress	3.27×10^{-5}	1.06×10^{-2}
GO:1901698	response to nitrogen compound	7.56×10^{-5}	1.11×10^{-2}
GO:1901576	organic substance biosynthetic process	7.89×10^{-5}	1.13×10^{-2}
GO:0009058	biosynthetic process	9.11×10^{-5}	1.28×10^{-2}
GO:0070887	cellular response to chemical stimulus	9.79×10^{-5}	1.35×10^{-2}
GO:1901362	organic cyclic compound biosynthetic process	1.03×10^{-4}	1.35×10^{-2}

GO:0031400	negative regulation of protein modification process	1.01×10^{-4}	1.36×10^{-2}
GO:1901700	response to oxygen-containing compound	1.24×10^{-4}	1.59×10^{-2}
GO:0031326	regulation of cellular biosynthetic process	1.28×10^{-4}	1.62×10^{-2}
GO:0019438	aromatic compound biosynthetic process	1.44×10^{-4}	1.68×10^{-2}
GO:0018130	heterocycle biosynthetic process	1.44×10^{-4}	1.71×10^{-2}
GO:0051248	negative regulation of protein metabolic process	1.43×10^{-4}	1.73×10^{-2}
GO:0051171	regulation of nitrogen compound metabolic process	1.41×10^{-4}	1.74×10^{-2}
GO:0061136	regulation of proteasomal protein catabolic process	1.55×10^{-4}	1.74×10^{-2}
GO:0065007	biological regulation	1.55×10^{-4}	1.77×10^{-2}
GO:0010033	response to organic substance	1.63×10^{-4}	1.77×10^{-2}
GO:0044283	small molecule biosynthetic process	1.67×10^{-4}	1.77×10^{-2}
GO:0010556	regulation of macromolecule biosynthetic process	1.69×10^{-4}	1.77×10^{-2}
GO:0010646	regulation of cell communication	1.62×10^{-4}	1.79×10^{-2}
GO:0071230	cellular response to amino acid stimulus	1.77×10^{-4}	1.82×10^{-2}
GO:0034976	response to endoplasmic reticulum stress	1.82×10^{-4}	1.85×10^{-2}
GO:0097190	apoptotic signaling pathway	1.85×10^{-4}	1.85×10^{-2}
GO:0023051	regulation of signaling	1.93×10^{-4}	1.89×10^{-2}
GO:0097327	response to antineoplastic agent	2.07×10^{-4}	2.00×10^{-2}
GO:0031331	positive regulation of cellular catabolic process	2.19×10^{-4}	2.08×10^{-2}
GO:0009266	response to temperature stimulus	2.23×10^{-4}	2.09×10^{-2}
GO:0032268	regulation of cellular protein metabolic process	2.49×10^{-4}	2.30×10^{-2}
GO:0009889	regulation of biosynthetic process	2.65×10^{-4}	2.37×10^{-2}
GO:0006979	response to oxidative stress	2.96×10^{-4}	2.38×10^{-2}
GO:0050794	regulation of cellular process	2.93×10^{-4}	2.39×10^{-2}

GO:0034654	nucleobase-containing compound biosynthetic process	2.64×10^{-4}	2.40×10^{-2}
GO:0035966	response to topologically incorrect protein	2.92×10^{-4}	2.41×10^{-2}
GO:1903364	positive regulation of cellular protein catabolic process	2.80×10^{-4}	2.44×10^{-2}
GO:0006986	response to unfolded protein	2.92×10^{-4}	2.44×10^{-2}
GO:1903506	regulation of nucleic acid-templated transcription	2.91×10^{-4}	2.46×10^{-2}
GO:1903050	regulation of proteolysis involved in cellular protein catabolic process	2.80×10^{-4}	2.47×10^{-2}
GO:2001141	regulation of RNA biosynthetic process	2.91×10^{-4}	2.50×10^{-2}
GO:0035556	intracellular signal transduction	3.54×10^{-4}	2.81×10^{-2}
GO:0010243	response to organonitrogen compound	3.70×10^{-4}	2.90×10^{-2}
GO:0009408	response to heat	3.85×10^{-4}	2.94×10^{-2}
GO:0006457	protein folding	3.90×10^{-4}	2.94×10^{-2}
GO:0048583	regulation of response to stimulus	3.97×10^{-4}	2.95×10^{-2}
GO:0006355	regulation of transcription, DNA-templated	3.83×10^{-4}	2.96×10^{-2}
GO:0009719	response to endogenous stimulus	4.11×10^{-4}	3.03×10^{-2}
GO:0033673	negative regulation of kinase activity	4.31×10^{-4}	3.10×10^{-2}
GO:0006469	negative regulation of protein kinase activity	4.31×10^{-4}	3.14×10^{-2}
GO:0031399	regulation of protein modification process	4.42×10^{-4}	3.14×10^{-2}
GO:0051246	regulation of protein metabolic process	4.59×10^{-4}	3.23×10^{-2}
GO:0034645	cellular macromolecule biosynthetic process	4.94×10^{-4}	3.43×10^{-2}
GO:0043604	amide biosynthetic process	6.43×10^{-4}	4.41×10^{-2}

Table S3. Top 10 Kyoto Encyclopedia of Genes and Genomes (KEGG) and Protein ANalysis THrough Evolutionary Relationships (PANTHER) pathway enrichment analysis of differentially expressed genes based on overrepresentation enrichment analysis using WebGestalt (WEB-based Gene SeT AnaLysis Toolkit). A FDR-adjusted *p*-value of <0.05 was considered as significant.

Database	Pathway	ID	*p*-Value	FDR *p*-Value	Gene
**KEGG**	**Protein processing in endoplasmic reticulum**	**hsa04141**	**1.97×10^{-5}**	**0.005968702**	**SEC24A,HSPH1,CRYAB,DDIT3,ERN1,HSPA4L,PPP1R15A,DNAJA1,HSPA1A,HSPA1B,HSPA1L,HSPA8,HSP90AA1,DNAJB1,ATF4,YOD1,DNAJC3,XBP1,HERPUD1**
**KEGG**	**MAPK signaling pathway**	**hsa04010**	**4.45×10^{-5}**	**0.006735185**	**DUSP10,GADD45A,DDIT3,DUSP1,DUSP2,DUSP5,FGF2,FOS,RASGRP3,CACNG4,NR4A1,HSPA1A,HSPA1B,HSPA1L,HSPA8,HSPB1,GADD45B,ATF4,NGF,MAP2K3,RELB,DUSP16,PLA2G4C,RAPGEF2**
KEGG	Legionellosis	hsa05134	0.000226104	0.022836467	CLK1,CXCL2,CXCL3,HSPA1A,HSPA1B,HSPA1L,HSPA8,HSPD1,CXCL8
KEGG	Glycosphingolipid biosynthesis	hsa00604	0.002133021	0.161576376	B4GALNT1,ST8SIA5,ST3GAL1,SLC33A1
KEGG	Tumor necrosis factor signaling pathway	hsa04668	0.003376261	0.204601423	CEBPB,FOS,CXCL2,CXCL3,ATF4,PIK3R2,MAP2K3,PTGS2,BCL3,CCL2,TNFAIP3
KEGG	Estrogen signaling pathway	hsa04915	0.005105695	0.257837584	FKBP4,FOS,HSPA1A,HSPA1B,HSPA1L,HSPA8,HSP90AA1,ATF4,PIK3R2,PLCB2
KEGG	Influenza A	hsa05164	0.007700788	0.285860861	NXF1,HSPA1A,HSPA1B,HSPA1L,HSPA8,DNAJB1,CXCL8,CIITA,OAS3,PIK3R2,NXT2,MAP2K3,DNAJC3,CCL2
**KEGG**	**Apoptosis**	**hsa04210**	**0.007811691**	**0.285860861**	**BCL2L11,GADD45A,DDIT3,ERN1,FOS,BIRC5,LMNB1,GADD45B,ATF4,NGF,PIK3R2,PMAIP1**
KEGG	Vascular endothelial growth factor signaling pathway	hsa04370	0.008771281	0.285860861	HSPB1,KDR,PIK3R2,PLCG2,PTGS2,VEGFA,PLA2G4C

KEGG	Antigen processing and presentation	hsa04612	0.009434352	0.285860861	HSPA1A,HSPA1B,HSPA1L,HSPA4,HSPA8,HSP90AA1,CIITA,TAPBP
**Panther**	**Oxidative stress response**	**P00046**	**0.001097967**	**0.12407022**	**DUSP10,DDIT3,DUSP1,DUSP2,DUSP5,MAP2K3,DUSP26,DUSP16**
**Panther**	**Apoptosis signaling pathway**	**P00006**	**0.003311246**	**0.187085413**	**BCL2L11,JDP2,FOS,HSPA1A,HSPA1B,HSPA1L,HSPA8,ATF3,ATF4,MAP2K3,RELB,BAG3**
Panther	Angiogenesis	P00005	0.011294128	0.425412143	FRS2,CRYAB,FOS,HSPB1,BIRC5,KDR,PAK3,PIK3R2,PLCG2,SFRP1,VEGFA,WNT7B,FZD5,PLA2G4C
**Panther**	**p38 MAPK pathway**	**P05918**	**0.020391561**	**0.499761758**	**DUSP10,GADD45A,DUSP1,HSPB1,MEF2D**
Panther	Transforming growth factor -beta signaling pathway	P00052	0.022113352	0.499761758	CITED2,GDNF,INHBA,SMAD7,MAP3K7CL,BMP2,SNIP1,GDF5,GDF15
Panther	5-Hydroxytryptamine degredation	P04372	0.046944994	0.884130711	ALDH1L2,MAOB,ALDH1A2
Panther	Vascular endothelial growth factor signaling pathway	P00056	0.063096336	0.969545511	CRYAB,HSPB1,KDR,PIK3R2,PLCG2,VEGFA
Panther	p53 pathway	P00059	0.06864039	0.969545511	GADD45A,GADD45B,GTSE1,PIK3R2,PMAIP1,CCNB1,CCNE1
Panther	Plasminogen activating cascade	P00050	0.149868654	1	MMP1,PLAU
Panther	Cholecystokinin receptor signaling map	P06959	0.178267898	1	FOS,CXCL2,NR4A1,HSPB1,CXCL8,MEF2D,PLAU,PTGS2,RGS2,SNAI1,KLF4

Table S4. Top 10 Kyoto Encyclopedia of Genes and Genomes (KEGG) and Protein ANalysis THrough Evolutionary elationships (PANTHER) pathway enrichment analysis of differentially expressed genes based on gene set enrichment analysis using WebGestalt (WEB-based Gene SeT AnaLysis Toolkit). A FDR-adjusted *p*-value of <0.05 was considered as significant.

Database	Pathway	ID	*p*-Value	FDR *p*-Value	Gene
KEGG	Legionellosis	hsa05134	0	0.002107365	CXCL2,CXCL3,HSPA1A,HSPA1B,HSPA1L,CXCL8
**KEGG**	**Protein processing in endoplasmic reticulum**	**hsa04141**	**0.001451379**	**0.039218176**	**HSPH1,CRYAB,DDIT3,ERN1,HSPA4L,PPP1R15A,DNAJA1,HSPA1A,HSPA1B,HSPA1L,HSP90AA1,DNAJB1,XBP1,HERPUD1**
KEGG	Salmonella infection	hsa05132	0.006861064	0.088606454	FOS,CXCL2,CXCL3,CXCL8
KEGG	Th17 cell differentiation	hsa04659	0.006791172	0.11075807	FOS,GATA3,HSP90AA1,IL6R,RORA
KEGG	Spliceosome	hsa03040	0.012216405	0.11980237	HSPA1A,HSPA1B,HSPA1L
**KEGG**	**MAPK signaling pathway**	**hsa04010**	**0.010355029**	**0.12613232**	**DUSP10,DDIT3,DUSP2,DUSP5,FOS,NR4A1,HSPA1A,HSPA1B,HSPA1L, GADD45B**
KEGG	Influenza A	hsa05164	0.030165913	0.21599424	HSPA1A,HSPA1B,HSPA1L,DNAJB1,CXCL8
KEGG	Longevity regulating pathway - multiple species	hsa04213	0.05090312	0.23114683	HSPA1A,HSPA1B,HSPA1L
KEGG	Chemokine signaling pathway	hsa04062	0.03902439	0.23675887	CCL26,CXCL2,CXCL3,CXCL8
KEGG	Amphetamine addiction	hsa05031	0.021630615	0.2479679	ARC,FOS,FOSB
**Panther**	**Apoptosis signaling pathway**	**P00006**	**0.0015625**	**0.01209643**	**FOS,HSPA1A,HSPA1B,HSPA1L,ATF3,BAG3**
Panther	Cholecystokinin receptor signaling map	P06959	0.00619195	0.03925464	FOS,CXCL2,NR4A1,HSPB1,CXCL8,PTGS2,RGS2,KLF4
Panther	Parkinson disease	P00049	0.039049234	0.114372276	HSPA1A,HSPA1B,HSPA1L
Panther	Oxidative stress response	P00046	0.108552635	0.21099159	DUSP10,DDIT3,DUSP1,DUSP2,DUSP5,MAP2K3,DUSP26
Panther	p38 MAPK pathway	P05918	0.2318105	0.41184673	DUSP10,GADD45A,DUSP1,HSPB1

Panther	Transforming growth factor -beta signaling pathway	P00052	0.42556635	0.6274077	CITED2,GDNF,INHBA,SMAD7,MAP3K7CL,BMP2,SNIP1,GDF15
Panther	Integrin signaling pathway	P00034	0.6068516	0.77451116	RND1,RND3,ACTN2
Panther	Wnt signaling pathway	P00057	0.89701897	0.89474875	MYCL,MYH3,PCDH8,PCDH18,MAP3K7CL,WNT3
Panther	p53 pathway	P00059	0.9716667	0.96805197	GADD45A,GADD45B,GTSE1,PIK3R2,PMAIP1,CCNB1,CCNE1
Panther	Angiogenesis	P00005	0.57471263	1	FRS2,CRYAB,FOS,HSPB1,BIRC5,KDR,PAK3,PIK3R2,PLCG2,SFRP1,VEGFA,WNT7B,FZD5,PLA2G4C
